# Measures of oral health-related quality of life in patients with bone graft and implant prosthetic rehabilitation at the anterior of mandible/maxilla among young and middle-aged adults: a retrospective pilot study

**DOI:** 10.1186/s40729-023-00496-w

**Published:** 2023-11-01

**Authors:** Kazuyuki Yusa, Shigeo Ishikawa, Nagiko Suzuki, Shunsuke Kunii, Naoki Okuyama, Tomoharu Hemmi, Mitsuyoshi Iino

**Affiliations:** 1 Department of Dentistry, Oral and Maxillofacial-Plastic and Reconstructive Surgery Faculty of Medicine, Yamagata University, 2-2-2 Iida-Nishi, Yamagata, 990-9585 Japan; 2 Department of Dentistry and Oral Surgery, Okitama Public General Hospital, Yamagata, 992-0601 Japan

**Keywords:** Bone grafting, Implant, Oral health-related quality of life (OHRQOL)

## Abstract

**Objectives:**

Dental implants are believed to contribute to improved masticatory function and oral health-related quality of life (OHRQOL), but the details remain unclear. The aim of this study was to evaluate the clinical outcomes of dental implant prosthetic rehabilitation after bone graft at the anterior mandible/maxilla based on OHRQOL, particularly in young and middle-aged patients.

**Methods:**

This retrospective study included 11 patients who received bone grafts at the anterior mandible/maxilla and dental implant surgery. Chewing function score and OHRQOL (using the Oral Health Impact Profile-14 questionnaire) were evaluated before and after completion of an implant-retained bridge or removable implant-supported denture.

**Results:**

Chewing function score tended to improve slightly after dental implant prosthetic rehabilitation, but none of the observed differences were significant. In the assessment of OHRQOL, relatively worse domain scores before completion of dental implant prosthetic rehabilitation were seen for Functional limitation, Psychological discomfort, and Psychological disability. Conversely, Social disability seemed relatively unaffected by tooth loss. All domain scores and total scores for items other than Physical disability and Social disability were significantly improved after completion of dental implant rehabilitation.

**Conclusions:**

Tooth loss in the anterior region may not significantly affect chewing function score, but can have a significant impact on OHRQOL. Bone grafts and dental implant prosthetic rehabilitation can resolve these problems, and the results of this study will benefit both patients and medical professionals in terms of treatment planning and informed consent.

## Introduction

Tooth loss mainly occurs due to dental caries and periodontal disease. Previous reports have mentioned that an increasing number of missing teeth negatively affects oral health-related quality of life (OHRQOL) [1]. Clinical studies, including in dentistry and oral and maxillofacial surgery, have mainly focused on the development of diagnoses, surgical results, and surgical procedures. The World Health Organization provides the following definition of health: “a state of complete physical, mental and social well-being and not merely the absence of disease or infirmity”. Recently, the health-related quality of life (HRQOL) of patients has increasingly caught the attention of medical professionals. Previous reports have found associations between rheumatoid arthritis, cardiovascular disorders, diabetes, and HRQOL [2, 3]. Some reports have also suggested a relationship between HRQOL and OHRQOL [4, 5], and attention has been focused on the relationship between OHRQOL and various oral diseases, such as periodontal disease [6], dental caries [7], and temporomandibular disorder [8]. Further, tooth loss is a dental disease that significantly affects OHRQOL [9]. Davis et al. mentioned that 45% of participants in their study felt unprepared for the effects of losing teeth [10]. They also reported that those patients were reluctant to accept the loss of teeth and were less self-confident as a result [11]. With tooth loss, the impact of anterior tooth loss on OHRQOL is particularly large. Imam et al. revealed that anterior missing teeth in particular had a strong impact on patients, exerting effects in terms of pain, physical disability, and psychosocial dimensions [12]. Furthermore, other reports have mentioned that patients with ≤ 12 missing teeth, but including some anterior teeth, were more likely to present with higher OHIP scores than those with ≤ 12 missing teeth, but no missing anterior teeth [13]. With advances in oral hygiene, such as brushing with fluoridated toothpaste, avoidance of cigarette smoking, and regular preventive dental check-ups [14] the prevalence of tooth loss has been on the decline in recent decades. The estimated global prevalence of total tooth loss decreased from 4.3% in 1990 to 4.1% in 2015 [15]. However, in patients with mandibular or maxillary tumors [16, 17], maxillofacial injuries, [18] or cleft lip and palate (CLP) [19], tooth loss is common even among young and middle-aged patients. In such cases, jawbone reconstruction using free vascularized bone grafts, iliac particulate cancellous bone marrow (PCBM) grafts, or bone block (BB) grafts are often performed to improve facial contours and allow the insertion of a dental implant. In recent years, this comprehensive therapeutic strategy has become widely applied and implant survival rates for these patients have reached an ideal level [20–22]. However, limited information has been accumulated regarding how this strategy affects the OHRQOL of patients.

The aim of this study was to evaluate the clinical outcomes of dental implant prosthetic rehabilitation after bone graft to the anterior mandible/maxilla based on OHRQOL, with a focus on young and middle-aged patients.

## Material and methods

### Patients

This retrospective study included patients who underwent bone grafting to the anterior mandible/maxilla and dental implant surgery in the Department of Dentistry, Oral and Maxillofacial Surgery at Yamagata University Hospital between 2009 and 2021. Approval was obtained from the ethics committee at Yamagata University Faculty of Medicine (Approval No. 2019-149). This study was a retrospective observational study, undertaken using the opt-out method of consent via our hospital website. Patients with both anterior and posterior graft sites were also included.

We retrospectively reviewed patient age, sex, number of implants, implant location, and duration of follow-up. Patients with graft sites confined to the posterior mandible/maxilla, age ≥ 65 years, or malignant tumors were excluded.

### Implant placement and prosthetic procedure

The implantation procedure was conducted in two stages. Once osseointegration of the dental implants had been obtained, the second stage of surgery was performed. Complete rehabilitation was defined as having occurred when the patient was successfully fitted with an implant-retained bridge or removable implant-supported denture. Implant survival time was measured from implant placement to either failure (removal) or last follow-up, and the implant survival rate was determined from the number of implants that remained as of last follow-up.

### OHRQOL and chewing function

OHRQOL before and after completion of the implant-retained bridge or removable implant-supported denture was evaluated using the Oral Health Impact Profile-14 (OHIP-14) questionnaire [23], which consists of seven domains: Functional limitation; Physical pain; Psychological discomfort; Physical disability; Psychological disability; Social disability; and Handicap. For each of these seven categories, the mean value is calculated from the values attributed to the two related questions. The higher the OHIP-14 score, the poorer the state of health for the patient.

Masticatory function was evaluated using a modification of the chewing function questionnaire established by Sato et al. [24] The sheet lists 20 foods and the chewing function score for each patient ranges from 0 to 100. The questionnaire was developed for complete denture wearers, and mean scores for denture wearers were 58.7 for those who were ‘satisfied’, 48.5 for ‘partly satisfied’, and 32.4 for ‘not satisfied’.

Mean score was calculated before and after the patient completed dental implant prosthetic rehabilitation.

### Statistical analysis

For comparisons of chewing function score and OHIP-14 before and after completion of dental implant prosthetic rehabilitation, the Wilcoxon signed-rank test was used. Values of *p* < 0.05 were considered statistically significant.

## Results

### Patients

**Fig. 1:**
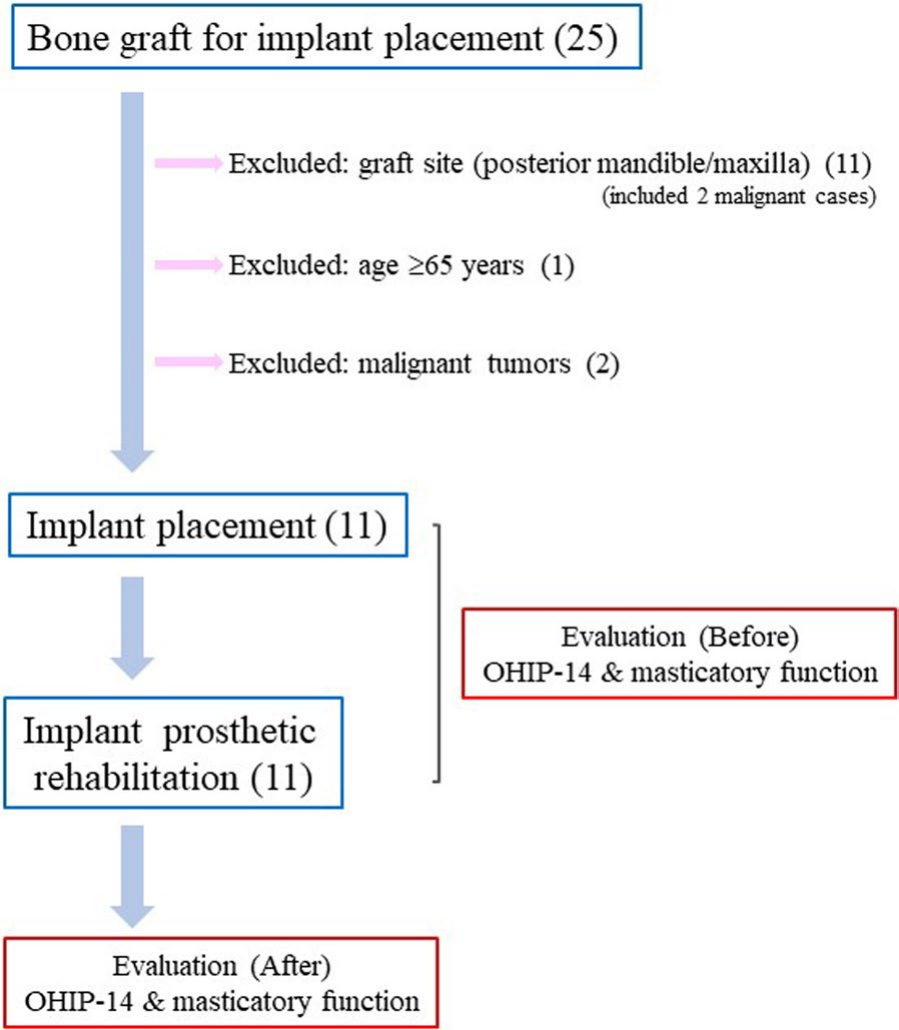
Flowchart for the selection of cases

Patients comprised 7 men and 4 women. Mean age at the time of bone graft was 31.64 ± 14.85 years (range, 17–60 years). The most common primary disease was traumatic injury (6 cases), followed by ameloblastoma (4 cases), and CLP (1 case). Graft sites were the maxilla in 6 cases and the mandible in 5 cases (Tables 1, 2, 3, Fig. 1**; blue arrow**). The mean number of remaining teeth (excluding third molars) before bone graft and dental implant insertion was 21.18 ± 5.04 (range, 8–26) (Tables 2, 3).

**Table 1:** Characteristics of the 11 patients

General characteristics	
Age (years); mean ± SD (range)	
31.64 ± 14.85 (17–60)	
≤ 19 years (*n*)	4
20–39 years (*n*)	3
≥ 40 years (*n*)	4
Sex	
Male (*n*)	7
Female (*n*)	4
Primary disease	
Traumatic injury (*n*)	6
Ameloblastoma (*n*)	4
Cleft lip and palate (*n*)	1
Grafted site	
Maxilla (*n*)	6
Mandible (*n*)	5

**Table 2:** Details of patients (maxillary cases)

Case	Age (years)	Sex	Primary disease	Bone graft	Teeth remaining	Dental implants	Schematic of bone graft and implant placement	Superstructure	Follow-up (months)
1	39	M	Traumatic injury	PCBM	23	5		IRB	119
2	22	M	Traumatic injury	BB	22	4		IRB	77
3	60	M	Traumatic injury	PCBM	21	3		IRB	39
4	19	M	CLP	PCBM	26	1		IRB	26
5	17	F	Traumatic injury	PCBM	25	2		IRB	24
6	17	F	Traumatic injury	PCBM	24	3		IRB	18

*CLP* cleft lip and palate, *PCBM* particulate cancellous bone marrow, *BB* bone block, *IRB* implant-retained bridge

**Table 3:** Details of patients (mandible cases)

Case	Age (years)	Sex	Primary disease	Bone graft	Teeth remaining	Dental implant	Schematic of bone graft and implant placement	Superstructure	Follow-up (months)
7	45	M	AMB	PCBM	8	8		IARPD	73
8	43	M	AMB	PCBM	17	5		IRB	66
9	43	M	AMB	PCBM	20	3		IRB	63
10	25	F	AMB	PCBM	24	3		IRB	56
11	18	F	Traumatic injury	PCBM	23	3		IRB	17

*AMB* ameloblastoma, *PCBM* particulate cancellous bone marrow, *IRB* implant-retained bridge, *IARPD* implant-assisted removable partial denture

### Bone graft and dental implant

Among the patients in this study, 10 of the 11 underwent jawbone reconstruction using PCBM. The remaining patient underwent bone graft using BB (Tables 2, 3).

A total of 40 implants were placed (1–8 implants/patient). Diameters of implants inserted into grafted areas ranged from 3.3 to 4.3 mm, and lengths ranged from 8.5 to 15 mm. After second-stage surgery, impression and bite-taking, an implant-retained bridge (10 patients) or implant-assisted removable partial denture (1 patient) was fabricated. The implant survival rate was 100% (40/40), with a mean follow-up of 52.54 ± 31.46 months (Tables 2, 3).

### OHRQOL and chewing function

**Fig. 2:**
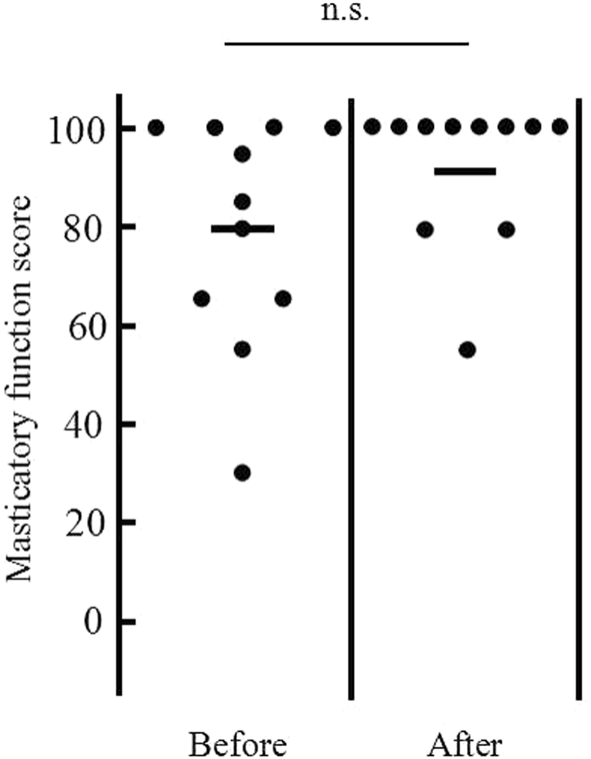
Chewing function score before and after completion of dental implant prosthetic rehabilitation. No significant differences are apparent between before and after completion of dental implant prosthetic rehabilitation

Table 4 and Fig. 2 summarize the results for OHRQOL and chewing function according to the OHIP-14 questionnaire and chewing function score.

**Table 4:** OHIP-14 scores before and after completion of implant prosthetic rehabilitation

OHIP-14	Before (mean ± SD)	After (mean ± SD)
Functional limitation	2.81 ± 0.98	1.18 ± 1.25*
Physical pain	2.45 ± 1.81	0.36 ± 0.67 **
Psychological discomfort	3.18 ± 2.14	1.00 ± 2.10*
Physical disability	1.18 ± 1.17	0.18 ± 0.40**
Psychological disability	2.82 ± 1.72	0.45 ± 1.21**
Social disability	1.55 ± 1.63	0.55 ± 1.04
Handicap	1.82 ± 1.83	0.55 ± 0.93**
Total	15.81 ± 7.93	4.18 ± 6.40**

*p < 0.05; **p < 0.01

The domain scores that were relatively worse before completion of dental implant prosthetic rehabilitation were those for Functional limitation (trouble pronouncing words and altered sense of taste) (before: 2.81 ± 0.98), Psychological discomfort (self-consciousness and feeling tense) (before: 3.18 ± 2.14) and Psychological disability (difficulty relaxing and feeling embarrassed) (before: 2.82 ± 1.72) (Table 4). Lower scores were seen for Physical disability (unsatisfactory diet and interrupted meals) (before: 1.18 ± 1.17) and Social disability (irritability and difficulty performing daily tasks) (before: 1.55 ± 1.63). All domain scores and total scores except those for social disability were significantly improved after completion of dental implant rehabilitation (Table 4).

Mean (± standard deviation) chewing function score was slightly higher after dental implant prosthetic rehabilitation, but no significant differences were identified (before: 80.00 ± 22.25; after: 92.28 ± 14.72) (Fig. 2).

## Discussion

The present study aimed to evaluate the influence of bone graft and dental implant prosthetic rehabilitation at the anterior mandible/maxilla on OHRQOL, with a focus on young and middle-aged patients. Our previous report revealed that in cases of malignancy, chewing function was apparently inhibited after tumor resection [25, 26]. These results were probably attributable to muscle tissue resection, jawbone resection, and tooth loss. Maeda et al. mentioned that masticatory function is controlled by various muscle movements [27]. In fact, other reports have investigated the electromyographic activity in patients who underwent marginal resection and found a difference in maximum voluntary clenching between the defect and non-defect sides [28]. On the other hand, in cases without malignancy, resection of muscle tissue is rare. Our previous report thus revealed no significant impairment in chewing function according to the　chewing function questionnaire [25]. Such trends are similar to the present results (Fig. 2), because patients in this study showed a relatively high number of remaining teeth and preserved muscle tissue (Tables 2, 3), so they could successfully chew almost all ingredients listed on the chewing function questionnaire before completion of bone graft and implant prosthetic rehabilitation. According to the results of OHIP-14, Physical disability (which consists of unsatisfactory diet and interrupted meals) had a relatively lower score even before implant prosthetic rehabilitation (Table 4). Social disability (which consists of irritable and difficulty performing daily tasks) was also relatively unrelated to teeth loss, and tendencies toward improvement following bone graft and implant prosthesis could be observed.

In contrast, we confirmed that patients in this study suffered from Functional limitation (before: 2.81 ± 0.98), Psychological discomfort (before: 3.18 ± 2.14), and Psychological disability (before: 2.82 ± 1.72). Tooth loss can adversely affect pronunciation, and indeed, a previous study reported that speech ability was significantly lower among individuals with fewer teeth [29]. In fact, none of the patients in the present study scored 0 (“never”) in the Functional limitation domain, especially for trouble pronouncing words. Furthermore, this study involved patients aged between 17 and 60 years, who were usually still employed or in school life, and presumably had many opportunities to come into contact with various other people. These backgrounds are also assumed to be one factor that led to psychological discomfort and psychological disability. Results from the OHIP-14 assessment apparently showed that implant prosthetic rehabilitation significantly improved scores in these domains. Although patients in this study had to undergo several surgical procedures (e.g., tumor resection, bone graft, and implant surgery), the fact that this series of treatments contributed to the improvement of OHRQOL was considered indicative of the legitimacy of the treatment.

We should note that the present study had several limitations. A key potential weakness of this study was the small sample size. To add to the evidence produced in our study, further longitudinal studies of larger numbers of participants are needed to strengthen and confirm our findings. A second limitation was that this study was based on subjective examinations. In the future, evaluations will need to be based on relationships with objective evaluations. Further, medium- and long-term observations will be necessary regarding relationships between implant survival and OHRQOL.

## Conclusions

The results suggest that dental implant prosthetic rehabilitation contributed to the improvement of OHRQOL. The results of this study will be of benefit to both patients and medical professionals in terms of treatment planning and obtaining informed consent.

## Data Availability

Not applicable.

## References

[1] Ide R, Yamamoto R, Mizoue T (2006). The Japanese version of the Oral Health Impact Profile (OHIP)–validation among young and middle-aged adults. Community Dent Health.

[2] Kosinski M, Keller SD, Ware JE, Hatoum HT, Kong SX (1999). The SF-36 Health Survey as a generic outcome measure in clinical trials of patients with osteoarthritis and rheumatoid arthritis: relative validity of scales in relation to clinical measures of arthritis severity. Med Care.

[3] Martínez-Castelao A, Gòrriz JL, Garcia-López F, López-Revuelta K, De Alvaro F, Cruzado JM (2004). Perceived health-related quality of life and comorbidity in diabetic patients starting dialysis (CALVIDIA study). J Nephrol.

[4] Heydecke G (2002). Patient-based outcome measures: oral health-related quality of life. Schweiz Monatsschr Zahnmed.

[5] Lee IC, Shieh TY, Yang YH, Tsai CC, Wang KH (2007). Individuals' perception of oral health and its impact on the health-related quality of life. J Oral Rehabil.

[6] Reynolds I, Duane B (2018). Periodontal disease has an impact on patients' quality of life. Evid Based Dent.

[7] Chaffee BW, Rodrigues PH, Kramer PF, Vítolo MR, Feldens CA (2017). Oral health-related quality-of-life scores differ by socioeconomic status and caries experience. Community Dent Oral Epidemiol.

[8] Pigozzi LB, Pereira DD, Pattussi MP, Moret-Tatay C, Irigaray TQ, Weber JBB (2021). Quality of life in young and middle age adult temporomandibular disorders patients and asymptomatic subjects: a systematic review and meta-analysis. Health Qual Life Outcomes.

[9] Anbarserri NM, Ismail KM, Anbarserri H, Alanazi D, AlSaffan AD, Baseer MA (2020). Impact of severity of tooth loss on oral-health-related quality of life among dental patients. J Family Med Prim Care.

[10] Davis DM, Fiske J, Scott B, Radford DR (2000). The emotional effects of tooth loss: a preliminary quantitative study. Br Dent J.

[11] Davis DM, Fiske J, Scott B, Radford DR (2001). The emotional effects of tooth loss in a group of partially dentate people: a quantitative study. Eur J Prosthodont Restor Dent.

[12] Imam AY (2021). Impact of tooth loss position on oral health-related quality of life in adults treated in the community. J Pharm Bioallied Sci.

[13] Batista MJ, Lawrence HP, de Sousa ML (2014). Impact of tooth loss related to number and position on oral health quality of life among adults. Health Qual Life Outcomes.

[14] da Franca Bandeira Ferreira Santos C, Godoy F, Menezes VA, Colares V, Zarzar PM, Ferreira RC, et al. School academic climate and oral health (tooth loss) in adolescents. PLoS One. 2020;15:e0233505. 10.1371/journal.pone.0233505 PMC7241719 32437411

[15] Kassebaum NJ, Smith AGC, Bernabé E, Fleming TD, Reynolds AE, Vos T (2017). Global, regional, and national prevalence, incidence, and disability-adjusted life years for oral conditions for 195 countries, 1990–2015: a systematic analysis for the global burden of diseases, injuries, and risk factors. J Dent Res.

[16] Fariña R, Alister JP, Uribe F, Olate S, Arriagada A (2016). Indications of free grafts in mandibular reconstruction, after removing benign tumors: treatment algorithm. Plast Reconstr Surg Glob Open.

[17] Hong J, Yun PY, Chung IH, Myoung H, Suh JD, Seo BM (2007). Long-term follow up on recurrence of 305 ameloblastoma cases. Int J Oral Maxillofac Surg.

[18] Kannari L, Marttila E, Thorén H, Snäll J (2022). Dental injuries in paediatric mandibular fracture patients. Oral Maxillofac Surg.

[19] Savoldelli C, Bailleux S, Chamorey E, Vandersteen C, Lerhe B, Afota F (2022). Impact of a new combined preoperative cleft assessment on dental implant success in patients with cleft and palate: a retrospective study. BMC Oral Health.

[20] Chatelet M, Afota F, Savoldelli C (2022). Review of bone graft and implant survival rate: A comparison between autogenous bone block versus guided bone regeneration. J Stomatol Oral Maxillofac Surg.

[21] Matsuo A, Hamada H, Takahashi H, Chikazu D (2019). Long-term structural changes and outcomes of implants in particulate cellular bone and marrow reconstructed jawbone. Clin Implant Dent Relat Res.

[22] Zavattero E, Ramieri G, Agrò G, Fasolis M, Garzino-Demo P, Borbon C (2021). Implant dental rehabilitation of fibula-free flap reconstructed jaws. J Craniofac Surg.

[23] Slade GD (1997). Derivation and validation of a short-form oral health impact profile. Community Dent Oral Epidemiol.

[24] Sato Y, Minagi S, Akagawa Y, Nagasawa T (1989). An evaluation of chewing function of complete denture wearers. J Prosthet Dent.

[25] Yusa K, Yamanouchi H, Yoshida Y, Ishikawa S, Sakurai H, Iino M (2017). Evaluation of quality of life and masticatory function in patients treated with mandibular reconstruction followed by occlusal rehabilitation with dental implants: a preliminary report. J Oral Maxillofac Surg Med Pathol.

[26] Yusa K, Hemmi T, Ishikawa S, Yamanouchi H, Kasuya S, Sakurai H (2020). Rehabilitation after maxillectomy in patients with implant-retained obturator: a preliminary report. Oral Surg Oral Med Oral Pathol Oral Radiol.

[27] Maeda M, Hirose M, Wada K, Kishimoto M, Akashi M, Kimoto A (2018). Elucidating the masticatory function and oral quality of life according to the range of mandibulectomy. J Oral Maxillofac Surg Med Pathol.

[28] Haraguchi M, Mukohyama H, Reisberg DJ, Taniguchi H (2003). Electromyographic activity of masticatory muscles and mandibular movement during function in marginal mandibulectomy patients. J Med Dent Sci.

[29] Steele JG, Sanders AE, Slade GD, Allen PF, Lahti S, Nuttall N (2004). How do age and tooth loss affect oral health impacts and quality of life? A study comparing two national samples. Community Dent Oral Epidemiol.

